# Student and staff experiences of digital dentistry in a Southwest Primary Care Dental School, UK

**DOI:** 10.1038/s41405-026-00458-5

**Published:** 2026-07-15

**Authors:** John Tredinnick-Rowe, Martha Paisi, Afsha Musa, Isabel Clark, Robert Witton, Andrew Sleigh

**Affiliations:** 1 Peninsula Dental Social Enterprise, Plymouth, UK; 2 Peninsula Dental School, University of Plymouth, Plymouth, UK; 3 Dental Public Health Training Programme, Southwest, UK

**Keywords:** Dental education, Dentistry

## Abstract

**Introduction:**

Digital dentistry (DD) is increasingly integrated into clinical practice, yet limited research explores its implementation within undergraduate dental education in the UK. This study examined student and clinical supervisor (CS) experiences of digital intraoral scanning (DIOS) in a primary care dental school.

**Methods:**

A qualitative study using semi-structured online interviews was conducted with 15 Bachelor of Dental Surgery (BDS) students and six CS in a dental school in Southwest England. Data were analysed using template analysis.

**Results:**

Participants viewed DIOS as essential for preparing graduates for contemporary practice, while maintaining competence in conventional techniques. Students described DIOS as engaging, low-stress, and beneficial for patient interaction, whereas CS highlighted technical challenges, variable staff digital literacy, and organisational constraints. Key barriers included limited equipment, restricted access, time pressures, and variability in supervisor training. Reported benefits included improved patient experience, accuracy, and enhanced learning for visually oriented students. Participants recommended increased access, structured training, and broader integration of digital technologies.

**Conclusions:**

DD teaching is positively received by students and valued by CS for preparing graduates for modern practice. However, successful integration requires investment in training, resources, and equitable access, alongside structured curriculum development to support consistent delivery.

## Introduction

While eHealth and related technologies are rapidly advancing across healthcare, research exploring their integration into dentistry remains limited. Existing studies tend to focus on the technical aspects of digital dentistry (DD), including diagnostic tools, scanning systems, design and manufacturing technologies, treatment planning and simulation, patient management, and artificial intelligence or machine learning applications [1]. In contrast, there is comparatively little research examining the intersection of dental education and DD.

Current literature [2] suggests that digitalisation has the potential to transform dental education by better preparing future dentists for contemporary clinical practice. Particularly promising are applications of augmented and virtual reality technologies, which may play a significant role in shaping the future of dental education.

One area of DD that has already been widely adopted in general dental practice is Digital Intraoral Scanning (DIOS) [3]. This technology replaces long-used analogue techniques for capturing the morphology of a patient’s dentition using impression materials such as alginate. Instead, it produces a digital three-dimensional scan of the patient’s dentition using specialised scanning devices and software capable of processing scan data into a usable 3D representation. Reported benefits of this technology over traditional techniques are speed of capture, accuracy, more efficient communication and logistics with the dental laboratory, and patient acceptance [3].

Introducing undergraduate teaching of new technologies is intended to equip the future dental workforce with both digital competencies and practical experience in operating alongside analogue techniques, reflecting the evolving landscape of clinical dentistry. Digital workflows are also anticipated to enhance treatment precision and reduce human error [4]. In the UK, dental schools are gradually implementing teaching of digital workflows to capture clinical information such as DIOS [5]; however, the effects of these initiatives remain poorly understood. Moreover, institutions have been slow to establish frameworks to evaluate new digital learning modules [6].

In this study, we aimed to explore the experiences and perceptions of both students and clinical supervisors (CS) with DD.

## Methods

### Context

Dental students at Peninsula Dental School (PDS) complete clinical placements at sites operated by Peninsula Dental Social Enterprise (PDSE). Both organisations collaborate to design and deliver the clinical curriculum for the Bachelor of Dental Surgery (BDS) programme. Ongoing curriculum review requires consideration of emerging clinical technologies, their benefits for patient care, and appropriate integration into undergraduate training to ensure graduates are prepared for contemporary dental practice.

**Fig. 1:**
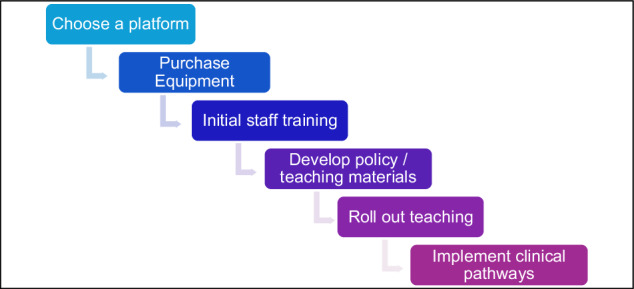
Implementation plan overview.

DIOS was identified as an important development in DD and a likely future clinical competency. Students were therefore provided with opportunities to understand clinical applications, compare digital and conventional techniques, and gain hands-on experience in a clinical setting. Measures were implemented to ensure students continued to gain sufficient experience with conventional impression techniques. An outline of the implementation plan is shown in Fig. 1.

**Fig. 2:**
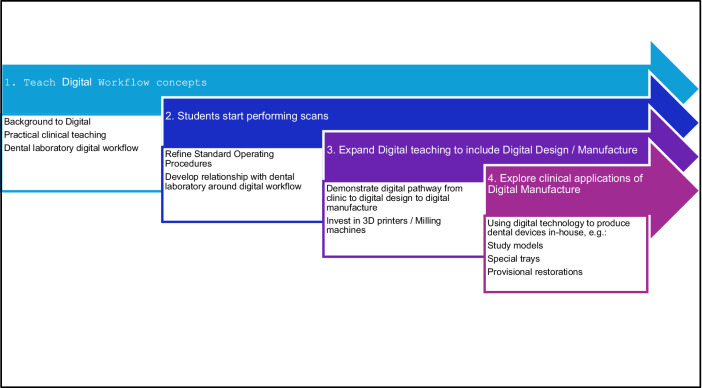
Roadmap for developing undergraduate DD Teaching.

PDSE purchased five DIOS devices in 2023 and an additional three in 2024. A staged implementation roadmap (Fig. 2) was developed, initially focusing on teaching digital workflow concepts and enabling supervised clinical scanning (Stages 1 and 2). Progression to later stages (3 and 4) was contingent on a subsequent feasibility review.

### Undergraduate teaching

Teaching was delivered in small groups (maximum 10 students per session) and included three components:

- **Plenary session:** Introduction to DIOS principles, comparison with conventional techniques, and overview of equipment use.
- **Hands-on clinical session:** Students worked in pairs to perform scans under clinical supervision. The activity was risk-assessed, informed consent was obtained, and alternative learning options were available.
- **Dental laboratory session:** Teaching on processing 3D scan data and key stages of the digital workflow, including digital manufacturing.

After completing these sessions, students were permitted to use DIOS as an alternative to conventional impression techniques in suitable clinical cases. Prerequisites were introduced to maintain balanced training, including completion of at least one case using traditional impressions before using DIOS.

### Study design

This qualitative study used semi-structured interviews to explore dental students’ and staffs' perceptions and experiences of digital workflows, capturing barriers, enablers, and implications for practice.

### Sampling strategy and recruitment

This study was conducted at PDS, University of Plymouth in Southwest England. Eligible participants included BDS students in years 4 and 5 and their CS, comprising GDC-registered dentists and dental therapists. Participants were recruited using a combination of convenience-based list recruitment and snowball sampling [7]. Course administrators emailed all eligible students, while the study was also promoted verbally at clinics, lectures, and through posters.

Participant recruitment was guided by the study aims and pragmatic considerations. Recruitment was bounded by structural constraints within the programme, including a finite student cohort and a defined population of 15 CS with experience of DD. Recruitment was also constrained by the academic teaching cycle and participant availability across cohorts, meaning the final sample reflected the total accessible population within the study period.

### Data collection and setting

Data were collected between June and December 2025 via semi-structured interviews conducted by JTR, using pilot-tested interview guides (Supplementary File 1). All but one interview was conducted online. Sessions were audio-recorded, auto-transcribed in MS Teams, and manually checked for accuracy. Participants discussed their experiences with a range of DD technologies, with a particular focus on intraoral scanners as part of undergraduate education.

### Data analysis

Data were analysed manually using template analysis, a structured form of thematic analysis employing hierarchical coding to organise themes [8, 9]. Anonymised transcripts were managed in Excel. Template analysis was chosen as it enables comparison of perspectives across stakeholder groups within a single analytical framework [9, 10].

JTR developed an initial coding template inductively following transcript familiarisation. Codes reflecting recurring patterns (e.g., clinical governance, staff relationships, and ways of working) were iteratively refined through comparison across student and CS interviews and organised hierarchically (Table 1). Cross-cutting themes related to DD delivery were identified through constant comparison [11].

**Table 1:** Themes and subthemes.

Theme	Subtheme
1**. Purpose of DD**	1.1 Preparedness for practice
	1.2 Pedagogy
	1.3 Fidelity
2**. Experience of DIOS**	
**3. Barriers for DIOS**	3.1 Standardisation and variability
	3.2 Space, access and equipment
	3.3 Time requirement to use DIOS
**4. Benefits of DIOS**	
**5. Recommendations**	5.1 Finding Time for DIOS
	5.2 Governance
	5.3 Gaps in provisions

A subset of transcripts and the developing coding template were reviewed by a second member of the research team (MP) to enhance analytical rigour. Differences in coding and theme development were discussed and resolved through consensus, and the coding framework was refined iteratively. Members of the wider research team contributed to the interpretation of findings through discussion of developing themes.

Reflexivity was maintained throughout, with ongoing consideration of researcher positionality and assumptions. Comparative analysis highlighted areas of agreement, divergence, and shared themes between staff and student participants.

### Ethical approval

All participants were provided with an information sheet and gave written informed consent through an online form (JISC) prior to the start of the study. Ethical approval for the study was granted by the Faculty of Health Research Ethics and Integrity Committee, University of Plymouth (ref: 5907).

## Findings

### Characteristics of participants

A total of 21 participants took part in the interviews, including six CS and 15 BDS students. Participant characteristics are shown in Table 2.

**Table 2:** Participant characteristics.

Participant Group (*n*)	Role	Gender
CS [6].	Dentist / Dental Therapist/Hygienist	4 male and 2 female
BDS [15].	BDS Year 4 BDS Year 5	5 male and 10 female

The themes and subthemes identified are presented in Table 1 and described below.

## Theme 1: The purpose of DD in undergraduate dental education

### Subtheme 1.1: Preparedness for practice

CS emphasised that while DIOS is not a new concept, recent technological developments have led to wider clinical acceptance. As one supervisor noted, “*we’ve actually had equivalents… going back a very long time. However, it seems to have become part of the zeitgeist*” (CS3).

Both supervisors and students viewed DIOS teaching as necessary to prepare students for contemporary dental practice. One CS commented that digital workflows are “*a rapidly growing aspect of dentistry which will be considered the ‘norm’ practice in due course*” (CS4). Similarly, students described DD as “*the future of the profession*” and part of “*moving with the times*” (BDS2). Students also highlighted the importance of remaining current in a rapidly evolving profession “*because dentistry is such a fast-developing career path and just like a field really, you sort of want to stay up to date with current practice”* (BDS4) and felt that prior exposure would increase confidence when entering practice, “*I wouldn’t feel scared. I’d feel pretty happy to go ahead*” (BDS11).

However, CS also emphasised the importance of maintaining competence in conventional techniques, noting that students should not lose essential “*wet skills*” such as impression taking and shade matching (CS6).

### Subtheme 1.2 Pedagogy

The approach to teaching DIOS emerged as an important consideration. Its inclusion in the curriculum was linked to future workforce needs, as DD “*is going to be part of teaching*” (CS2). However, DIOS was described as requiring “*a different skill set… a different type of skill*” (CS2), raising concerns about staff digital literacy.

Supervisors highlighted challenges related to teaching expertise: “*If you’re teaching something, you want to be familiar with it… The reality is, very few people who’ve got lots of experience with the digital stuff*” (CS3). Consequently, effective implementation requires whole-team training, as “*the whole team needs to have education and training on it… everybody’s all singing from the same hymn sheet*” (CS7), particularly to avoid inequities in delivery across sites and cohorts.

DIOS teaching was intentionally integrated alongside existing methods rather than replacing them: “*I’ve… kept in place all the previous teaching, and then we’ve layered on the digital stuff… so that you get both ways of looking at it*” (CS3), supporting different learning styles. Students responded positively to the teaching format, describing it as “*something a bit different… new and… fun*” (BDS2), though many requested additional or repeat sessions to reinforce learning and prevent skill decay (BDS1).

### Subtheme 1.3 Fidelity

Students and supervisors highlighted the importance of making DIOS clinics reflect real workplace practice. One CS suggested extending access to dental hygienists and therapists to increase fidelity, though current DIOS teaching focuses on dental students, as fixed prosthodontics falls outside the scope of other professionals. Some students wished to practice on patients rather than peers, with a few arranging supervised sessions themselves to gain “*the confidence of them being able to use it [hardware] on a live patient*” (BDS2).

## Theme 2: The experience of DIOS

Most students found learning the hardware straightforward: “*the sessions run quite smoothly, and people did pick it up relatively quickly*” (BDS5), and described the environment as “*fairly low stress, low pressure*” (BDS2). Students practised on each other following formal instruction, though readiness to use DIOS on patients varied, from “*I’d happily book in treatment to use it if I could*” (BDS3) to needing additional sessions (BDS13).

Students noted a risk of skill loss: “*I think we just need a bit more experience… I haven’t had any experience doing it [IOS] with any patients*” (BDS12); “*it takes a bit of practice… it would be better if it was a bit more accessible to use*” (BDS10). Peer-to-peer practice served as useful simulation, but with limited real-world efficacy.

CS highlighted technical challenges and skill acquisition issues: “*It’s not massively user-friendly*” (CS7); “*I felt like I was talking to the computer more than the patient”* (CS4). They also raised concerns that infrequent access to DIOS hardware could lead to “de-skill” (CS1).

## Theme 3: Barriers to implement DIOS

CS cautioned that DIOS may risk students relying on technology at the expense of fundamental clinical skills: “*my only concern with digital is the tendency for people to start chasing after the big, shiny new toy and forget all the other things that have gone before, which remain highly relevant*.” (CS2) Students may fail to critically evaluate clinical decisions, instead applying digital solutions uncritically. Teaching emphasised this, noting that DIOS software uses machine learning, but clinicians must still compare scans with clinical observations.

Consequently, CS suggested that DIOS is most appropriate in later years of training: “*would it [DIOS] be wasted if it was with the year ones… once you start getting to year four and year five, they are more competent, and they could probably start to see how they could bring that into their treatment plan and use it*” (CS1).

### Subtheme 3.1: Standardisation and variability

DIOS implementation is difficult to standardise, raising potential equity issues due to differences in supervisor training and system heterogeneity. Not all supervisors were equally familiar with the software: “*some of them had varying degrees of familiarity with it [the software]”* (BDS2). Students also noted that supervisors were sometimes learning alongside them: “*some of the supervisors… were sort of training with us, kind of getting to know how to use it as well*” (BDS5).

With over 100 supervisors, achieving consistent training was challenging: “*getting everybody together… trying to calibrate your style… you’re going to inevitably end up with some variability*” (CS7). However, this variability was also seen as beneficial, allowing students “*to see different ways of doing things… the richness of different supervisors working slightly differently is a value to them*” (BDS5), contributing to fidelity and diversity in learning.

### Subtheme 3.2: Space, access and equipment

Organisational integration was as important as equipment quality or cost: “*a lot of dental schools operate on a Dental hospital model… the dental hospital is like an entity in itself*” (CS2). DIOS systems are expensive and require ancillary equipment, with limited resources affecting student access: “*there weren’t enough anterior and posterior tips*” (BDS7).

Physical space and IT integration also posed challenges: “*we’ve only got 2… downstairs… not easily accessible… three bays upstairs… you’ve got to faff around*” (BDS11). Students reported difficulties in booking and coordinating scanner use: “*if we were able to… see who is currently in those chairs… then go back to your patient and ask can you do this date*” (BDS14).

### Subtheme 3.3 Time requirement to use DD

In an educational setting, DIOS setup can be time-consuming, reducing efficiency: “*they only get set up when you say… took about 40 minutes*” (BDS9); “*some of my patients can’t stay that long… we just have to take the impression*” (BDS14). While scanning is generally faster than conventional impressions, delays in preparation affect workflow and raise fidelity considerations.

## Theme 4: Benefits of DIOS

Students highlighted patient-centred advantages, including cleaner procedures, reduced gag reflex, and better aesthetics: “*from the patient perspective, I imagine they prefer the scanner… impressions… are quite messy*” (BDS2); “*aesthetic reasons*” (BDS4); “*cleaner*” (BDS6). DIOS was also seen as quicker, more accurate, and easier to correct: “*quick turnarounds to the lab… no argument about an impression*” (BDS11); “*if they go wrong, you shouldn’t have to start from scratch*” (BDS5). 3D imaging enhanced patient engagement: “*show a patient the interactive… screen*” (BDS15).

Supervisors observed rapid skill acquisition: “*I was pleased with how quickly the students were able to pick up the [intraoral] scanners”* (CS2). However, learning benefits may vary by style; visually oriented learners adapt easily, while others prefer hands-on tactile experience: “*like to have something in their hands… physically touch something*” (CS3).

## Theme 5: Recommendations

Both CS and students offered suggestions to improve DIOS implementation. CS focused on system-level issues, while students emphasised regular access and hands-on use.

### Subtheme 5.1: Finding time for DIOS

With a rigid curriculum, innovative approaches are needed to allow student practice. One CS suggested using unscheduled downtime: “*sometimes patients don’t turn up… Is there a way that it [DIOS] could be available… they could use it*?” (CS1). Self-directed sessions were proposed to maintain engagement.

### Subtheme 5.2: Governance

CS highlighted the importance of early planning for governance and long-term costs: “*governance processes take time to get resolved… engagement of everyone… critical right from the outset*” (CS2); “*think about… ongoing costs, and… when this thing becomes obsolete*” (CS3). Students requested more frequent access: “*I kind of wish they’d use it [DIOS] more regularly… one per bay that was set up for the day*” (BDS9), and suggested written protocols for unavailable equipment.

### Subtheme 5.3: Gaps in provisions

CS recommended expanding the digital workflow curriculum, including in-house 3D printing: “*produce certain items ourselves*” (CS2); “*3D printing for special trays*” (CS7). Students requested broader access to technologies, including AI software, note-taking tools, 3D printing, OPG machines, and 3D anatomy displays.

## Discussion

From a systems perspective, students and CS in this study agreed on the importance of DIOS for preparing graduates for future practice but differed in emphasis. CS focused on implementation, governance awareness, and long-term skills development, whereas students prioritised immediate clinical and patient experience. Similar divergence between students and educators has been reported previously [12], suggesting that digital education should extend beyond technical skills to include understanding of system limitations, governance, and data management. Teaching non-technical aspects of DD in a structured manner may be more efficiently achieved in a non-clinical environment where students can learn and discuss these aspects of DD as a group, rather than opportunistically in a clinical setting. This supports the chosen teaching methodology, where students received an initial plenary session covering both technical and non-technical aspects of DD before progressing to practical teaching in the clinical environment.

The perceived benefits of DIOS were also shaped by the educational environment. Although DIOS is associated with time efficiency in clinical practice [13], teaching contexts introduced additional demands such as equipment booking, setup, and troubleshooting, which reduced this advantage. While these challenges may reflect authentic clinical realities associated with digital workflows, it remains unclear whether exposure to such issues supports preparedness for practice or unnecessarily hinders learning during early skill acquisition [14].

Decisions about teaching fidelity were also important. While simulation traditionally precedes patient care, allowing students to scan each other provided a practical, high-fidelity and time-efficient transition to clinical use for a low-risk procedure. Variability in supervisor experience was expected given the novelty of DIOS and differences between digital systems. Although variation in teaching approaches may enrich learning, it also raises issues of consistency and equity across cohorts. The identification of institutional “digital champions” may support more consistent delivery.

Current literature has primarily focused on students’ attitudes toward the introduction of DD curricula; however, the evidence base remains limited and geographically dispersed. For example, Zitzmann et al [2]. reviewed 20 studies exploring digital surface mapping among dental students, examining the optimal timing for introducing these elements into the curriculum and their effects on student performance. Similarly, quantitative research involving prosthodontic students in Germany demonstrated positive student perceptions regarding the implementation of DD within the preclinical curriculum [15]. In contrast, the present study explored both student and staff experiences and perspectives on the introduction of DD. Findings from CS suggested that students may become overly reliant on technology at the expense of developing fundamental clinical skills. Consequently, DIOS may be more appropriately introduced during the later stages of undergraduate training.

Although university curricula must evolve to reflect advances in DD and contemporary professional competencies, previous research examining educational needs relating to DIOS has similarly suggested that the technology may be most beneficial to students in the advanced years of undergraduate study. At this stage, students may be better positioned to integrate fundamental clinical knowledge with the practical application of digital technologies into routine patient care [16].

At a strategic level, collaboration between dental schools to develop a shared digital curriculum may improve consistency while supporting innovation. However, DIOS integration remains at an early stage, constrained by limited curriculum space, the continued requirement to teach conventional techniques, equipment costs, and infrastructure considerations [17, 18]. Financial, IT, and data governance requirements therefore remain significant considerations for wider implementation.

### Strengths and limitations

This is, to our knowledge, the first study to explore student and CS perspectives within a primary care education setting where DIOS has been embedded into comprehensive patient care, which enhances the novelty of our findings. This contrasts with usual models in which DIOS education is delivered within specific teaching blocks, such as prosthodontics [19]. A potential benefit of this integrated approach is the opportunity to consider the application of DIOS across different dental modalities, for example fixed prosthodontics, orthodontics and implant planning.

The study also provides rich qualitative insight into both student and CS perspectives on the integration of DIOS within such a setting. Inclusion of multiple stakeholder groups enabled comparison across educational and supervisory roles, enhancing the depth and relevance of the findings. The use of semi-structured interviews and inductive template analysis supported a nuanced exploration of implementation challenges and educational value within a real-world primary care training environment.

However, several limitations should be considered. As the study was conducted within a single dental school, transferability to other institutional contexts with differing resources, curricula, or levels of digital integration may be limited. Potential selection bias may also be present, as individuals with a particular interest in DD may have been more likely to participate. The focus on early-stage implementation of DIOS means that experiences may change over time as familiarity, infrastructure, and training develop.

### Opportunities

The findings highlight several opportunities for developing DD education.

A balanced approach combining simulation and clinical exposure may support both patient safety and preparation for practice while maintaining curriculum efficiency.

DIOS may also enhance students’ experience in other areas of dental education, such as practicing operative techniques, by providing students with real-time, high-fidelity feedback on their work.

Digital workflows provide scope for enhanced interdisciplinary and interprofessional learning, integrating prosthodontics, radiology, dental technology, and biomaterials more effectively.

Finally, rather than replicating rapidly evolving commercial technologies, dental schools may focus on developing foundational digital competencies and digital literacy in later years of training, supporting graduates’ adaptability and lifelong learning.

## Conclusion

DIOS was positively received by students and valued by CS as a means of supporting graduates for contemporary dental practice. Effective integration of DD into the undergraduate curriculum requires balancing simulation and clinical exposure, alongside ensuring equitable access and consistent delivery across cohorts. While implementation is associated with practical challenges, including setup time and variable equipment reliability, participants also perceived these as reflective of real-world clinical conditions. Strategic opportunities include the development of shared curricula, strengthening interdisciplinary learning, and fostering digital literacy and lifelong learning to support graduates in adapting to evolving technologies.

## Supplementary information

Topic Guides for Students and Staff
