# Supplementary material for: Student and staff experiences of digital dentistry in a Southwest Primary Care Dental School, UK
Source: BDJ Open. 2026 Jul 15;12:73. doi: 10.1038/s41405-026-00458-5 (PMC13373226; doi:10.1038/s41405-026-00458-5)
Supplement: Supplementary file 1 — Topic Guides for Students and Staff [file 41405_2026_458_MOESM1_ESM.docx]

## Topic Guide for Students

Version 3. 18.12.24

**Consent Process**

- Step 1: Talk the participants through the process.
- Step 2: Give participants the opportunity to ask questions.
- Step 3: Re-confirm consent.

**Questions**

**Q1**: Why do you think more digital dentistry has been introduced to your learning?

**Q2** This is a new way of working. What has your experience been like (experiences positive and negative)?

- **PROMPT:** How is it different from what you were doing before?
- **PROMPT:** What works well? What are the challenges?
- **PROMPT:** Is there anything else you’d like added to the delivery?

**Q3:** What implications do you think learning this skill will have for your professional practice?

**Q4**: Have there been any barriers/challenges in the sessions?

**Q5:** Is there anything that worked well that you want to highlight?

**Q6:** What do you think the impact of the new approach will be on patients and dentistry overall?

**Q7:** Are there any specific changes to the digital dentistry elements of the course you’d like to see?

- **Prompt**: Do you have any recommendations for optimising the digital dentistry elements of the course?

**Debrief**

- **Reassurance of confidentiality and anonymity in all reports and publications arising from the project**

**END**

## Topic Guide for Staff

Version 5. 17.01.25

**Consent Process**

Step 1: Talk the participant through the process.

Step 2: Give participants the opportunity to ask questions.

Step 3: Re-confirm consent.

**Questions**

**Q1**: Why do you think a digital dentistry component was introduced to the BDS curriculum?

**Q2:** This is a new way of working. What was the implementation process like?

- **PROMPT:** How is your role different from what you were doing before?

**Q3:** Have there been any adaptations or adjustments to the delivery since you started?

- **PROMPT:** To explore experiences so far (positives, negatives), challenges in the implementation, what they found helpful,

**Q4:** Have any gaps in support been identified so far?

**Q5:** As a clinical supervisor, what has been the main barrier to delivering digital dentistry so far?

**Q6:** As a clinical supervisor, what has helped you deliver the digital dentistry elements so far?

**Q7:** More widely, what do you think the impact has been on students or patients?

**Q8:** Do you have any recommendations for optimising the further rollout of digital dentistry?

**Debrief**

- **Reassurance of confidentiality and anonymity in all reports and publications arising from the project**

**END**
